# Efficient Degradation of Feather by Keratinase Producing *Bacillus* sp.

**DOI:** 10.1155/2013/608321

**Published:** 2013-11-05

**Authors:** P. Jeevana Lakshmi, Ch. M. Kumari Chitturi, V. V. Lakshmi

**Affiliations:** Department of Applied Microbiology, Sri Padmavati Mahila Visvavidyalayam, Tirupati 517502, Andhra Pradesh, India

## Abstract

Keratinase producing microorganisms are being increasingly utilized for degradation and recycling of poultry feather waste. Two native strains BF11 (*Bacillus subtilis*) and BF21 (*Bacillus cereus*) degrading keratin completely were characterized. The native strains produced more than 10 KU/mL of enzyme. Strain improvement resulted in isolation of MBF11 and MBF21 from BF11 and BF21 isolates, respectively. Optimization of nutritional and physical parameters of these MBF isolates at laboratory scale increased the overall keratinase activity by 50-fold resulting in a yield of 518–520 KU/mL. Fermentation media designed with starch as carbon source and soya bean meal as nitrogen source supported high levels of enzyme production. The optimum conditions for enzyme production were determined to be pH 8.5 and temperatures of 45–55°C for MBF11 and 37°C for MBF21, respectively. Culture filtrate showed a significant increase in the amounts of cysteine, cystine, methionine, and total free amino acids during the fermentation period. The ratio of organic sulphur concentration was also considerably higher than that of the inorganic sulphate in the culture filtrate suggesting the hydrolysis of disulphide by the isolates.

## 1. Introduction

Feather is generated in bulk quantities as a by-product in the poultry industry globally. It is a very rich source of protein with *β*-keratin constituting 91% of feather protein. The presence of keratin makes feather recalcitrant to most common proteases like trypsin, pepsin, papain, and so forth, thus slowing down its degradation process in nature [1]. Typically, each bird has up to 125 gm of feather and with more than 400 million chickens being processed every week worldwide, the daily accumulation of feather waste reaches five million tons [2]. The bulk of feather waste is poorly recycled in nature and has limited utility due to the chemically unreactive nature of keratin. Conventionally, this waste has been converted into feed supplement, resulting in feed of poor quality which is nonviable economically [3]. Thus, recycling of this by-product is neither profitable nor environmentally friendly. The disposal of this waste is a global environmental issue leading to pollution of both air and underground water resources [4].

In recent years, feather treated with microbial keratinase is attracting wide attention with several applications. Keratinase-treated feather is increasingly considered as a viable source of dietary protein in food and feed supplements, as the enzyme-treated end product retained high nutritive value. Keratinases are projected to generate a potential worldwide market similar to other proteases. Diverse groups of microorganisms are reported to produce keratinase like fungi (*Doratomyces microsporus*, *Alternaria radicina*, *Trichurus spiralis*, *Aspergillus* sp., *Rhizomucor* sp., *Absidia* sp., *Stachybotrys alba, etc.*), actinomycetes (*Streptomyces pactum, S. alvs, S. thermoviolaceus, S. fradiae, Thermoactinomyces candidus etc.*), and several bacterial species (*Fervidobacterium islandicum, Pseudomonas aeruginosa*, *Microbacterium *sp*.,* and many species of* Bacillus *including *Bacillus licheniformis *and *B. pumilus*) earlier [2, 5–8]. Among bacteria, *Bacillus* spp. appear to be promising for keratinase production on commercial scale [4, 5, 9–11]. However, the full commercial potential of keratinases is yet to be realized. Application of rDNA technology to increase the yield of keratinase had limited success. Ker A gene from *Bacillus licheniformis* PWD-1 responsible for keratinase production though cloned and expressed in *Bacillus subtilis, E. coli, *and *Pichia pastoris* has the issues of solubility of the expressed product and/or stability of the cloned genes which are yet to be resolved [12]. At present, the major focus in this field still rests in identifying novel isolates with high keratinase activity and improving the yield using conventional and r-DNA approaches, in addition to optimizing the physical and nutritional parameters to maximize keratinase yield. The present study reports the use of these approaches to characterize and improve keratinase production by two *Bacillus* sp. isolates obtained from poultry farms and feather dumps in and around Tirupati, India.

## 2. Materials and Methods

Samples were collected from the feather dumps, poultry manure, and fresh poultry litter from local poultry farms in and around Tirupati, Andhra Pradesh, India. For enriched isolation of sporulating bacteria, the samples were heat treated at 80°C for 20 minutes. Keratinase producing organisms were isolated by feather baiting technique on nutrient agar plates [13]. Potential keratinolytic isolates were reisolated as pure cultures and confirmed for keratinase production in basal medium and complete media [14]. Keratinase enzyme was assayed by azokeratin assay adopting the method of Lin et al. [4]. Azokeratin substrate was prepared by adopting the method of Riffel et al. [15].

### 2.1. Identification of Bacterial Isolates

Identification of the keratinolytic bacterial isolates was carried out by determining morphological, cultural, biochemical, and genetic characters. The colony morphology, pigment production, and diffusibility were tested on the nutrient agar. Microscopic morphology of the isolates was studied by Gram staining using bright field [16, 17] and by negative staining with 1% phosphotungstic acid for electron microscopic examination [18]. The optimum cultural characterization of the two isolates was determined by testing range of growth temperature (from 4°C to 65°C), pH (5.5 to 8.5), salinity (1–10%), oxygen requirement, and so forth. Further biochemical characterization was carried out adopting standard tests as described in Collee et al. [19].

16S ribosomal typing was carried out for characterization of the isolates to the species level and phylogenetic analysis. The genomic DNA was extracted by using bead beater (Omni model 8) using 2 mL polypropylene Beadbeater vial with 0.5 g each of 0.1 mm and 3.0 mm silica-zirconium beads at 4 m/s for 40 seconds [20]. 16S rDNA amplification was carried out using PB36 as forward primer (AGR GTT TGA TCM TGG CTC AG (R=G/M=A/C K=T/G A)) and PB38 as reverse primer (GKT ACC TTG TTA CGA CTT) [21]. The primer pair, dNTP, Taq polymerase, and other buffers were purchased from Invitrogen. DNA sample was purified from gel slices using PCR-DNA purification kit (Qualigens) followed by sequencing of the PCR product in a 313 OXL capillary DNA sequencer (Applied Biosystems). The gene sequence obtained was BLAST searched to fish out homologous sequences. The CLUSTAL W aligned sequences were analyzed using neighbor-joining method [22] and phylogenetic analysis was carried out using Treecon package version 1.3b for Windows. The sequences are deposited in GenBank with accession numbers EU360724.1 (BF11) and EU360725.1 (BF21).

### 2.2. Maximizing the Production of Keratinase

The two selected native isolates were subjected to treatment with a combination of physical (UV light) and chemical (hydroxylamine hydrochloride (HA) and ethyl methyl sulphonate (EMS)), mutagens adopting the method of Eisentadt [23]. The organisms were exposed to sublethal doses of the mutagen for different time intervals. The screening for mutants with higher protease activity was carried out on milk agarose medium and isolates showing significant increase in zone of clearing were selected to quantify keratinase [4]. Subjecting isolates to stepwise mutagenesis with physical and chemical mutagenic agents resulted in isolation of MBF11 and MBF21 from BF11 and BF21 isolates, respectively. These isolates were used for optimization of keratinase production.

### 2.3. Optimization of Nutritional and Physical Parameters

For optimization of nutritional parameters, basal medium was supplemented with nine different carbon sources individually at concentration range from 0.2% to 1% and five nitrogen sources in concentration range between 0.2 and 1.5 percent. Fermentation was carried out for seven days and the keratinase production was assayed at 24 hr. intervals. The optimum carbon and nitrogen source and concentration thus identified were used to design the optimum fermentation media. The effect of substrate concentration on production of keratinase for improved MBF isolates was studied using optimized media with concentration between 0.25% and 1.5%. The physical parameters like temperature (in the range of 25–55°C), pH (in the range of 5.5–9.5), and agitation were optimized for maximum yield of keratinase production for the improved isolates. Degradative products profile of keratinase fermentation like sulphur rich compounds cysteine, cystine [24], and methionine [25], total inorganic sulphur released [26], and amount of free amino acids [27] was estimated using the methods specified, respectively.

## 3. Results and Discussion

**Figure 1:**
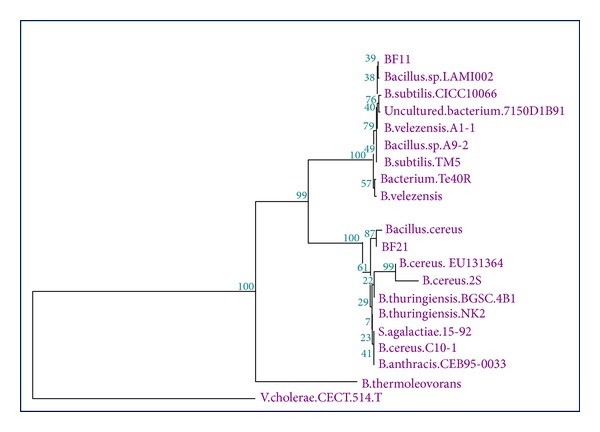
Phylogenetic analysis of 16S rRNA gene sequence of BF isolates.

A total of 80 samples were analysed for potential keratinase producing organisms. 400 keratinophilic microorganisms were isolated, out of which 120 isolates exhibited keratinolytic activities. Among these, 55 isolates showed significant (>50%) degradation of feather substrate, while 27 isolates exhibited moderate activity (with 25–50% degradation). 38 isolates degraded the feather poorly with less than 25% degradation. The isolates showing moderate to high keratinolytic activity were tested for the production of keratinase enzyme in basal media. Two isolates BF11 and BF21 exhibiting maximum keratinase were selected and the keratinase production was compared in three liquid media. Both the isolates showed lower keratinase production in Luria broth and nutrient broth tested (with maximum of 10.2 KU/mL) as compared to basal media where the activity reached 12.8–14.7 KU/mL. There was no significant variation in biomass production in three media. Thus, the results suggested that basal medium supported better yield of keratinase. Characterization of the two isolates was carried out by determining morphological, cultural, and biochemical characters. BF11 and BF21 isolates were aerobic, motile, and sporulating rods which were unable to grow below 10°C. The organisms were moderately thermo-tolerant (with growth up to 55°C) and halotolerant (tolerating up to 7% salt concentration). The BF isolates showed good growth between pH ranges of 5.5 to 9.5, with optimum at 8.5. In biochemical characters, both organisms were catalase positive and produced H_2_S. BF11 did not utilize lactose, glycerol, and galactose where arabinose, lactose, xylose, and galactose were not utilized by BF21 (Table 1). Based on the biochemical characters, the two isolates were grouped under the genus *Bacillus*. In the 16S rDNA typing and phylogenetic analysis (Figure 1), BF11 was designated as *Bacillus subtilis* and BF21 as *Bacillus cereus.*

**Figure 2:**
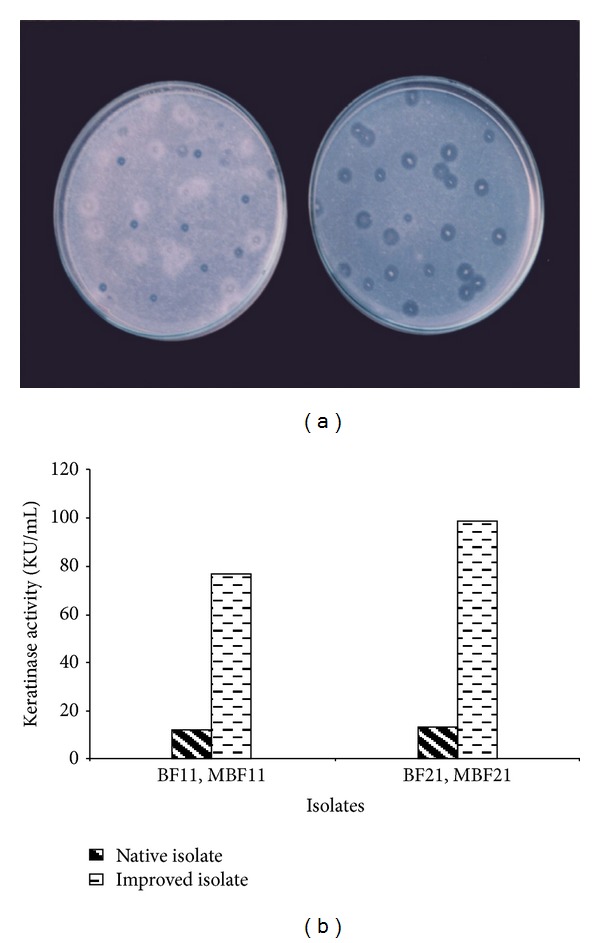
(a) Screening of improved isolates on milk agar plates. (b) Comparison of keratinase activity between native and improved isolates.

**Figure 3:**
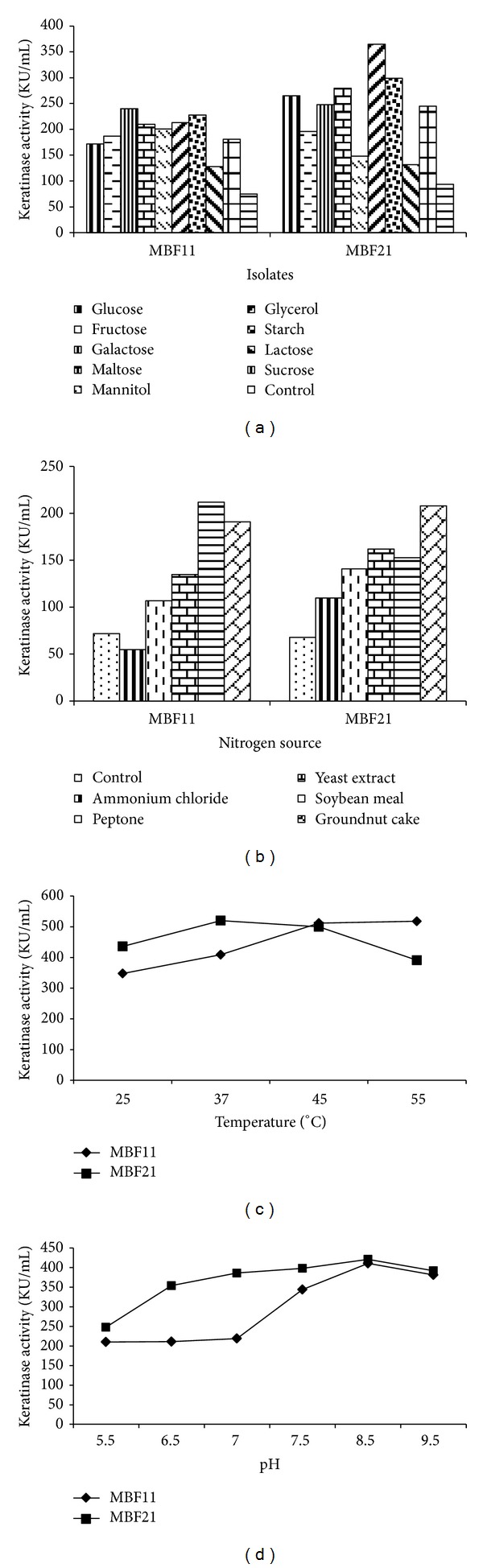
Optimization of nutritional and physical parameters for keratinase production by MBF11 and MBF21. Effect of (a) carbon sources, (b) nitrogen sources, (c) temperature, and (d) pH on keratinase production.

Optimization of parameters of fermentation was carried out using basal media for MBF11 and MBF21 strains obtained from BF11 and BF21, respectively, by stepwise mutagenesis. The maximum keratinase yield of the improved strains reached 75–90 KU/mL (Figures 2(a) and 2(b)). Influence of the nine different carbon sources on the fermentative production of keratinase for the two MBF isolates was compared and the results are shown in Figure 3(a). Starch and galactose (228–240 KU/mL) supported the highest production of keratinase followed by fructose, maltose, and sucrose (187–210 KU/mL) for BF11. Glycerol and starch (365–299 KU/mL) were the best among the nine carbon sources tested for MBF21 followed by glucose, galactose, maltose, and sucrose, with the yields ranging between 245 and 280 KU/mL. Supplementation of carbon source in basal media resulted in significantly enhanced keratinase production as compared with control basal media for both the isolates. There was a 1.5- to 2-fold increase in keratinase activity by increase in starch concentration between 0.5 and 1%. Further increase in starch concentration up to 2.5% did not yield any significant enhancement in keratinase production. Our results identified starch, galactose, and glycerol as good carbon sources in the order mentioned. Starch was not observed to be an ideal carbon source for keratinase production [1] though it has been reported to be good source for alkaline protease production by *B. licheniformis *and *B. polymyxa* [9]. Glucose also supported a higher level of keratinase compared to the control basal media though it was lower than starch. Glucose was observed to repress keratinase production in several bacteria such as *Bacillus licheniformis, Bacillus *sp. FK 46, and partly *Thermoactinomyces candidus* [11, 12, 28]. Glucose had no effect on keratin degradation with *Bacillus *sp. FK46 [10]. However, presence of glucose as carbon source was found to stimulate the keratinase production in *Streptomyces* sp. *Stenotrophomonas* sp., *Microbacterium *sp., and so forth, as well as in fungi [8, 29]. Thus, starch being the cheapest and most viable carbon source emerged is suitable for design of media for the test organisms. 

The results of effect of nitrogen source on keratinase production are shown in Figure 3(b). Among the nitrogen sources tested, soybean meal was found to be the best nitrogen source for MBF11 (212 KU/mL), whereas the maximum yield for MBF21 (207 KU/mL) was obtained with groundnut cake supplementation. The enzyme production increased up to 2-fold with the increase in concentration of organic nitrogen sources from 0.2 to 1%, after which a plateau was observed for both the isolates. Yeast extracts were the other nitrogen sources supporting enzyme production. Inorganic nitrogen source in the form of NH_4_Cl had no significant effect on the production of keratinase for the MBF isolates, with the yield being either lower than or comparable to controls. Supplementation of soyabean meal was observed to enhance the production of the alkaline protease as well as keratinase in *Bacillus *sp. [12]. However, it was not found to support good keratinase yield with *B. licheniformis *[4]. Optimisation of C/N ratio resulted in designing fermentation media where basal medium was supplemented with 1% concentration each of relatively inexpensive raw materials, starch and SBM/GNC. This medium supported optimum production of keratinase and is also suitable for bringing down the overall cost while scaling up the production.

Keratinase production was observed only in the presence of the feather substrate, indicating that it was mainly inducible. Increase in substrate concentration up to 10 mg/mL was found to enhance keratinase production with both the MBF isolates. Further increase in substrate concentration upto 20 mg/mL did not exhibit any significant effect on keratinase yield. The production of keratinase occurred in late exponential phase or in the stationary phase of microbial growth with corresponding degradation of feather substrate in the period. A 3-4-fold difference in enzyme yield was observed between the agitated condition (380 and 464 KU/mL) and static conditions (120 and 124 KU/mL). In general, production of keratinase requires high aeration and submerged conditions with the exception of keratinase reported from thermophilic anaerobic bacteria where enzyme production was favored by static anaerobic conditions [7, 30]. The optimum temperature for MBF11 was 45–55°C indicating the thermotolerant nature of the enzyme whereas that for MBF21 was 37°C (Figure 3(c)). The results of pH effect on production of keratinase in the range of 5.5–9.5 (Figure 3(d)) indicated that an increase in pH from 5.5 to 8.5 resulted in a gradual increase in keratinase yield with both strains' optimum being 8.5. In terms of growth, the organisms showed less growth at pH 4.5 and above 8.5 but the growth was comparable between pH 5.5 and 8.5 indicating that the influence seen was not due to differences in the total biomass. Alkaline pH has been observed to enhance the production of keratinase enzyme in earlier reports too [12]. The yield of enzyme with use of designed media and under optimized conditions increased to 518–524 KU/mL units for MBF organisms, thus resulting in 50-fold enhancement compared to the native isolates. This yield was significantly higher than several of the keratinase producing bacterial species reported earlier but was comparable with *Bacillus licheniformis* PWD1 [12, 31].

**Figure 4:**
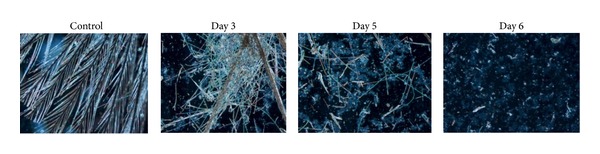
Stages of feather degradation (magnified 40 times).

Degradation of feather substrate was found to be associated with significant increase in pH of the medium to alkalinity, thus serving as an indicator for the efficiency of degradation. A good correlation was observed between increases in the concentration of degradative products and the levels of degradation of the substrate. The tendency of the medium to turn alkaline has been attributed to deamination reactions leading to production of ammonia from protein, peptides, and amino acids during keratin degradation. Increase in alkalinity has also been observed to favor further rapid enzymatic attack of keratin resulting in higher levels of keratinolysis [15]. The pattern of feather degradation was observed at various stages of degradation them under microscopes (Figure 4). Degradation of feather shaft was achieved by the fifth day and the substrate was completely degraded into white powdery mass by the seventh day. The degradation started with the adhesion of the bacteria to the feather. The organisms were found to penetrate the quill region of feather first with the separation of barb which was broken down into powdery gelatinous mass. This was followed by spread to shaft region leading to its rupture and disintegration of the shaft to needle like structures by the sixth day leading to complete degradation of feather (Figure 4).

**Figure 5:**
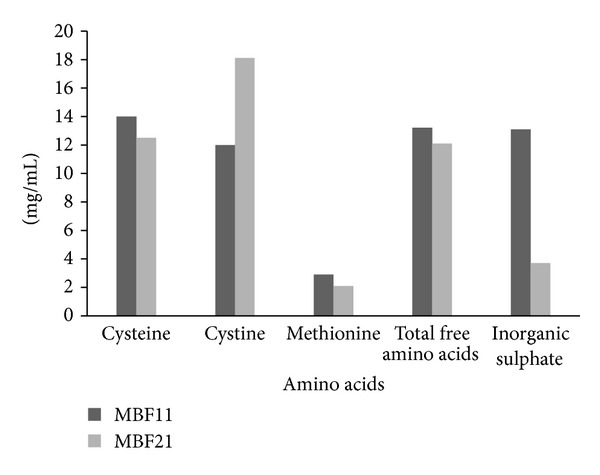
Comparison of analysis of culture filtrate of MBF11 and MBF21.

The analysis of culture filtrate showed a significant increase in the amounts of cysteine, cystine, methionine, and total free amino acids during the fermentation period (Figure 5). There was a gradual increase in the concentration of cysteine from 2.5 *μ*g/mL on the first day to 12.5–14 *μ*g/mL by the fifth day for MBF11 and MBF21. The cystine levels reached peak by 5-6 days with 18.1 *μ*g/mL for MBF21 isolates and 12.8 *μ*g/mL for MBF11, after which a decline was observed. The levels of soluble amino acids like cysteine and cystine released by the BF isolates were higher than earlier reports where 5 *μ*g to 16 *μ*g/mL was observed in different studies [6, 8]. Though 35 *μ*g/mL cystine was reported to be released on degradation of hair by *Microsporum gypseum* by Malviya et al. [32], the period of incubation was 50 days. The ratio of inorganic sulphur, peptides/amino acids, or other sulfhydryl compounds liberated from degradation is considered as index of degree of keratinolytic activity of an organism [33]. The ratio of sulphur compounds released by MBF isolates showed that the inorganic sulphate concentration was significantly lower than the organic sulphur in the culture filtrate for the isolates. The presence of the products of sulphitolysis such as peptides, cysteine, cysteine, and sulphate in the culture filtrate of MBF isolates confirmed the disulphide breakdown.

## 4. Conclusions

The thermotolerant nature of keratinase with optimum activity at alkaline pH has a definite advantage thereby increasing the versatility and the application potential. 70% of the total organic degradation products released between 96 and 120 hours constituted sulphur containing amino acids, suggesting a good potential application of these organisms as well as cheaper means for production of commercially important by-products. Thus, the present study is a step forward in the process of production of indigenous keratinase enzyme.

## Figures and Tables

**Table 1:** Morphological and biochemical characterization of BF isolates.

Character	Test	Isolates	
		BF11	BF21
Growth at (°C)	4	−	−
	10	−	−
	27	+	+
	37	+	+
	45	+	+
	55	+	+
	65	−	−
	4	−	−

Growth at different pH	4.5	−	−
	5.5	+	+
	6.5	+	+
	7.5	+	+
	8.5	+	+
	9.5	+	+
	10.5	−	−

Biochemical tests	Indole	−	−
	MR	−	−
	VP	+	+
	Citrate	+	+

Oxygen requirement		A	A

Spore staining		+	+

Motility		+	+

Sugar utilization	Arabinose	−	−
	Glucose	+	+
	Sucrose	+	+
	Lactose	−	−
	Fructose	+	+
	Maltose	+	+
	Mannitol	+	+
	Xylose	−	−
	Glycerol	+	+
	Galactose	−	−
	Starch	+	+

Enzymatic activity	Oxidase	−	−
	Catalase	+	+
	Urease	−	−
	Nitrate reduction	+	+
	Gelatinase	+	+
	Caseinase	+	+
	Amylase	+	+
	*β*-Galactosidase	−	−

Salt Tolerance (%)	2	+	+
	5	+	+
	7	+	+
	10	−	−
